# Construction of a Glycolysis-related long noncoding RNA signature for predicting survival in endometrial cancer

**DOI:** 10.7150/jca.50413

**Published:** 2021-01-01

**Authors:** Yuan Jiang, Jie Chen, Jingxian Ling, Xianghong Zhu, Pinping Jiang, Xiaoqiu Tang, Huaijun Zhou, Rong Li

**Affiliations:** 1 Department of Obstetrics and Gynecology, Nanjing Drum Tower Hospital, Nanjing Medical University, Nanjing 210008, China; 2 Department of Obstetrics and Gynecology, Nanjing Drum Tower Hospital, Affiliated Hospital of Nanjing University Medical School, Nanjing 210008, China; 3 Department of Gynecology, The First Affiliated Hospital of Nanjing Medical University, Nanjing 210029, Jiangsu Province, China

**Keywords:** Glycolysis, lncRNA, TCGA, endometrial cancer, risk signature, nomogram.

## Abstract

**Background:** long noncoding RNA (lncRNA) has been widely studied and understood in various cancer types. However, the expression profiles of glycolysis-related lncRNA in endometrial cancer (EC) have poorly been reported.

**Methods:** In this study, we retrieved the “Glycolysis” gene list from Molecular Signatures Database (MSigDB) and screened prognostic glycolysis-related lncRNA using The Cancer Genome Atlas (TCGA) Uterine Corpus Endometrial Carcinoma (UCEC) RNA-seq dataset. Then, TCGA UCEC patients were randomly divided. Lasso algorithm and multivariate cox regression analyses were then performed to further select hub prognostic lncRNA and to develop a prognostic signature. The efficacy of the signature was also evaluated in the TCGA EC cohort. Moreover, we constructed a nomogram to predict EC patient outcomes.

**Results:** Univariate cox analysis identified thirty-six glycolysis-related lncRNA correlated with EC patient prognosis. Among them, five lncRNA were further selected as hub lncRNA that mostly relate to EC patient outcomes, which are AL121906.2, BOLA3-AS1, LINC01833, AC016405.3, and RAB11B-AS1. A prognostic signature was then built based on the expression and coefficiency of five lncRNA. The efficacy of the signature was validated in part of and the entire TCGA EC cohort. In addition, the risk signature could precisely distinguish high- and low-risk EC patients and predict patient outcomes. The nomogram exhibited absolute concordance between the predictions and actual survival observations.

**Conclusions:** The glycolysis-related lncRNA signature model and the nomogram may provide a new perspective for EC patients outcome prediction in clinical use.

## Introduction

Malignancies are not only genetic diseases but also metabolic diseases. Since the "Warburg effect" was first uncovered by OttoWarburg, which is a remarkable phenomenon explicitly elucidating the transition of glycometabolism from oxidative phosphorylation to anaerobic glycolysis in cancer cells, our understanding of why tumors develop metabolic phenotypes that differ from adjacent, nonmalignant tissues have significantly been improved^1^-^3^. The reprogramming of cell metabolism is a hallmark of various cancer types, including endometrial cancer^3^. Endometrial cancer (EC) ranks the third most common female reproductive malignant tumors, leading to approximately 90,000 global deaths each year. Metabolic disorders, including glucose and lipid metabolism, are high-risk factors for endometrial cancer. It is reported that overweight or obese women, especially those with diabetes or high blood cholesterol level, have a remarkably increased risk of developing endometrial cancer than common individuals^4^. At the genetic level, overexpression of the glucose transporter GLUT6 in EC was highly correlated with the malignant cell phenotype and survival^5^. Besides, Mori Y et al. reported that ALDH-mediated activation of glycolysis promoted the paclitaxel resistance in endometrial cancer^6^. These studies suggest that metabolic changes, especially glucose metabolism, may promote the initiation and progression of endometrial cancer.

Long noncoding RNA (lncRNA) is a kind of conservative non-coding RNA with over 200 nucleotides in length that does not have any protein-coding ability. Aberrant expression of lncRNA plays a pivotal role in regulating multiple and complex biological processes, including cell proliferation, metabolism, and differentiation in many cancer types^7^, Chen et al reported that HIF-1α-stabilizing long noncoding RNA (HISLA) that transmitted from tumor-associated macrophages (TAMs) extracellular vesicle (EV) could effectively promote the aerobic glycolysis and apoptotic resistance of breast cancer cell^8^. Likewise, Lin found that LINK-A (long intergenic non-coding RNA for kinase activation) activated normoxic HIF1α signaling, promoting the glycolysis reprogramming and tumorigenesis in triple-negative breast cancer^9^. However, lncRNA involved in metabolism reprogram in endometrial cancer has yet not fully elucidated. Liu et al. recently performed GSEA analysis of dysregulated genes in the TCGA EC cohort and results showed that the "Glycolysis" gene set was highly enriched^10^. Therefore, in this study, we focused on the relationship between glycolysis-related lncRNA and endometrial cancer treatment. We performed cox and lasso regression analysis to select prognostic glycolysis-related lncRNA and subsequently constructed a prognostic risk model and a nomogram for TCGA Uterine Corpus Endometrial Carcinoma (UCEC) patients' OS evaluation. This study may open up new ideas for the treatment of EC.

## Materials and methods

### EC patients' gene expression and clinical data collection

541 EC patients' RNA-seq data (the FPKM format) and corresponding clinical information including survival time and status, patient age, clinical stage, tumor grade, and histology, and lymph nodes status were downloaded from The Cancer Genome Atlas (TCGA) database (https://cancergenome.nih.gov/) and the cBio Cancer Genomics Portal (cbioPortal, http://cbioportal.org), respectively^11^.

### Glycolysis-related genes and lncRNA selection

Glycolysis-related genes were retrieved from the gene set "HALLMARK_GLYCOLYSIS" in Molecular Signatures Database (MSigDB). The relationship was calculated based on the expression value between lncRNA and glycolysis-related genes. lncRNA with Spearman's correlation coefficient with an absolute value of >0.4 and p < 0.001 were set for further analysis.

### Identification of the prognosis associated glycolysis-related lncRNA in TCGA-UCEC patients

Univariate Cox regression analysis was performed to identify prognostic associated glycolysis-related lncRNA. Hazard Ration (HR) < 1 means better overall survival outcomes (OS) whereas HR > 1 presents worse OS. Genes with P < 0.05 were considered as independent prognostic associated glycolysis-related lncRNA and used to construct the lncRNA risk score. Besides, the expression level of prognostic associated glycolysis-related lncRNA from each patient and between cancerous and normal samples was displayed via "pheatmap" and "ggplot" R package, respectively.

### Construction of lasso Cox regression model for key lncRNA related to the prognosis of EC

A total of 541 TCGA-UCEC patients were randomly divided into the training and the testing cohort. The Least Absolute Shrinkage and Selection Operator (LASSO) analysis is preferred to select a small number of features from a large number of candidates with a certain lambda parameter. We selected hub prognostic associated glycolysis-related lncRNA by performing LASSO and multivariate Cox regression analysis via the “glmnet” R package. The risk score for each EC patient was computed as follows: 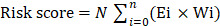 where N is the number of prognostic lncRNA, E_i_ is the expression value of lncRNA_i_, and W_i_ is the multivariate coefficient for lncRNA_i_. Patient survival status, death time, and lncRNA expression condition were unfolded via the "pheatmap" and "survival" R packages. In addition, Kaplan-Meier curve analysis, the time-dependent receiver operating characteristic (ROC) curve as well as the area under curve (AUC) analyses were used to evaluate the sensitivity and specificity of the lncRNA risk score for survival prediction.

### Validation of the predictive efficacy of the prognostic lncRNA risk signature

The predictive efficacy of the risk signature was measured in the testing and the entire cohort. The risk score of patients in each cohort was calculated and ranked. The discrepancy of the different subgroups was then displayed regarding patients' survival status and survival time, as well as lncRNA expression. KM curve and ROC curve analysis were performed as well. Besides, we conducted principal component analysis (PCA) regarding the risk signature and other gene profiles to measure its classifying efficacy.

### Total RNA extraction of clinical tissues and quantitative real-time PCR (qRT-PCR) analysis

Ten EC tissues and 10 normal endometrial tissues were obtained from patients at the Department of Gynecology, Nanjing Drum Tower Hospital. Normal tissues were obtained from individuals who underwent a hysterectomy due to endometrial-irrelevant diseases. All samples were immediately snap-frozen in liquid nitrogen and stored at -80°C until further analysis. Each individual provided informed consent, and this study was approved by the Ethics Committee of Nanjing Drum Tower Hospital. Total RNA of tissues was extracted using TRIzol reagent (Vazyme, Nanjing, China) and TaqMan Reverse Transcription kit (Applied Biosystems) with random hexamer primers was used to reverse-transcribe cDNAs corresponding to the mRNAs of interest. 2×SYBR Green qPCR Master Mix (Selleck, Shanghai, China) was used for qRT-PCR and the housekeeping gene GAPDH was used for normalization of the data before calculation using the ΔΔCt method. The primers used are listed in Table S1.

### Evaluation of the clinical characteristics of lncRNA risk signature

TCGA-UCEC patients who lacked any detailed clinical index including patient age, clinical stage, tumor grade and histology, and lymph nodes status were removed, and the clinical and gene expression data of the remaining low- and high-risk subgroup patients were compared. Uni- and multivariate Cox regression analysis and ROC curve analysis regarding clinical indexes and risk scores were then performed to evaluate the independency and the predictive efficacy of the risk model. Besides, the clinical characteristics of hub lncRNAs of the risk signature were also measured.

### Comprehensive clinical nomogram building

In the light of patients' risk scores and clinical features including age, grade, weight, histology, stage as well as lymph node status, we built a comprehensive prognostic nomogram to estimate EC patients' survival probability based on the TCGA entire set via the “rms” R package.

## Results

### Identification of a list of prognostic associated glycolysis-related lncRNA

**Figure 1:**
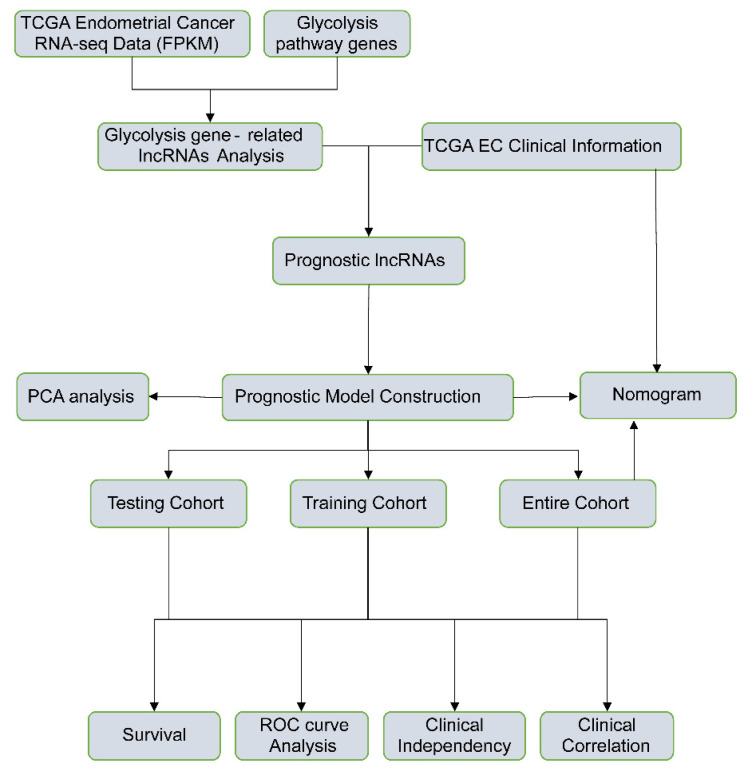
The flow chart of this study in identifying a prognostic glycolysis-related lncRNA signature.

**Figure 2:**
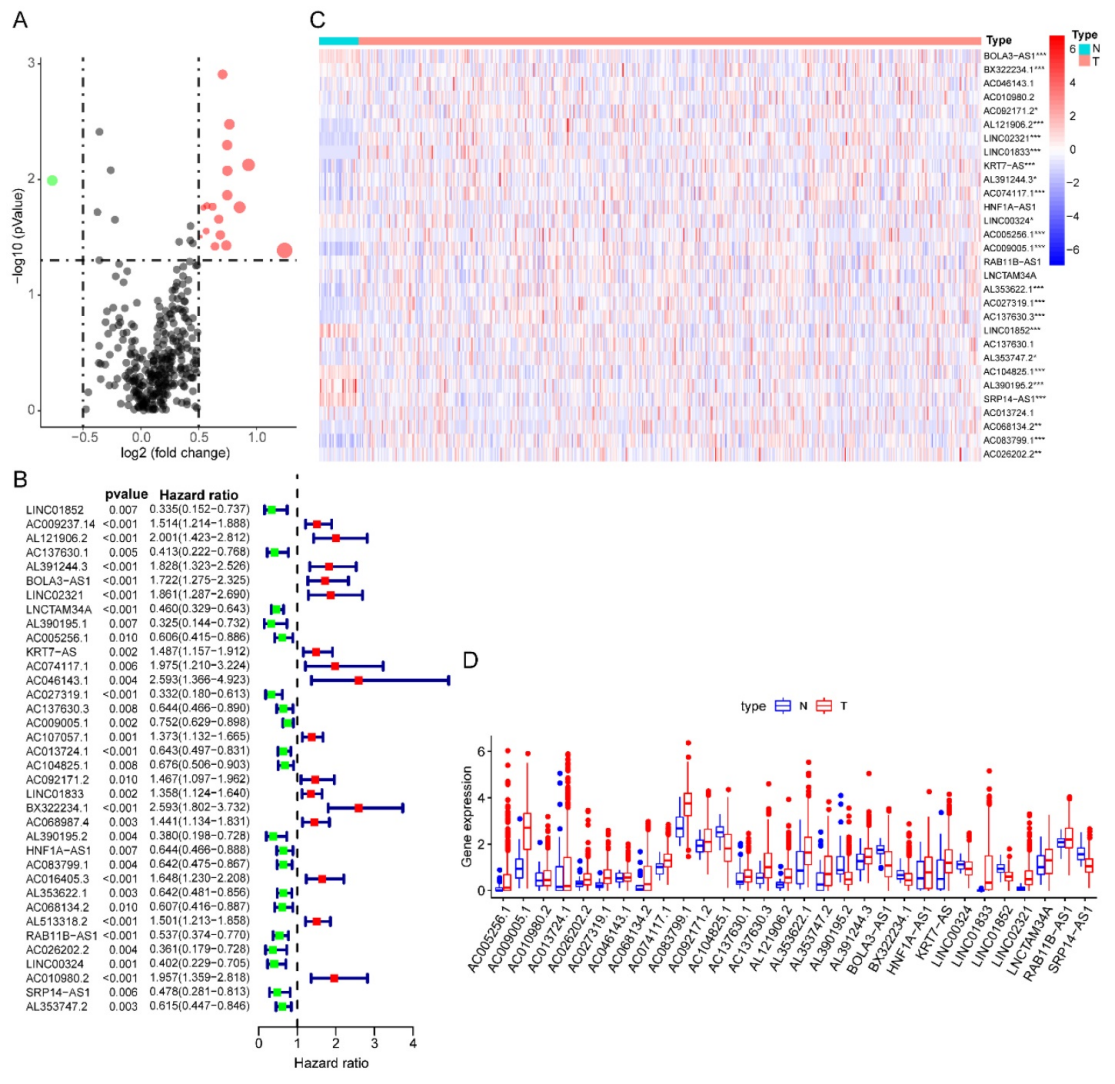
** The expression profiles of prognostic glycolysis-related lncRNA between TCGA endometrial cancer (EC) and normal tissues. (A)** Volcano plot of glycolysis-related lncRNA in EC and normal samples of the TCGA dataset. The horizontal axis indicates the log2 (fold change [FC]) and the vertical axis indicates the -log(P-value). Red dots and the green dots represent over- and down-expressed lncRNAs, respectively (P-value<0.05 and |log2(FC)|>1). **(B)** Univariate Cox regression identified 36 lncRNAs correlated to EC patients' outcomes. **(C)** Heat map of the 36 lncRNAs in the entire CGA EC cohort. Red and blue indicate higher expression and lower expression, respectively. * represents <0.05, ** represent <0.01, ***represent <0.001. **(D)** Box plot of the expression of the lncRNAs between cancerous and normal tissues. Red and blue boxes indicate cancerous and normal tissue, respectively.

The detailed flow chart for the prognostic predictive model construction in this study was shown in Figure 1. From the gene set "HALLMARK_GLYCOLYSIS" in MSigDB, we extracted 200 genes involved in glycolysis and gluconeogenesis. Then, according to the correlation efficiency and probability cut-off value, a total of 522 lncRNAs were considered as glycolysis-related lncRNA. Univariate Cox regression analysis further identified 36 lncRNA significantly correlated to EC patients' OS (Figure 2B and Table 1). The expression profile of 36 prognostic associated glycolysis-related lncRNA was presented in the heatmap and box plot (Figure 2C-D).

### Construction of a five prognostic associated glycolysis-related lncRNA risk signature

**Figure 3:**
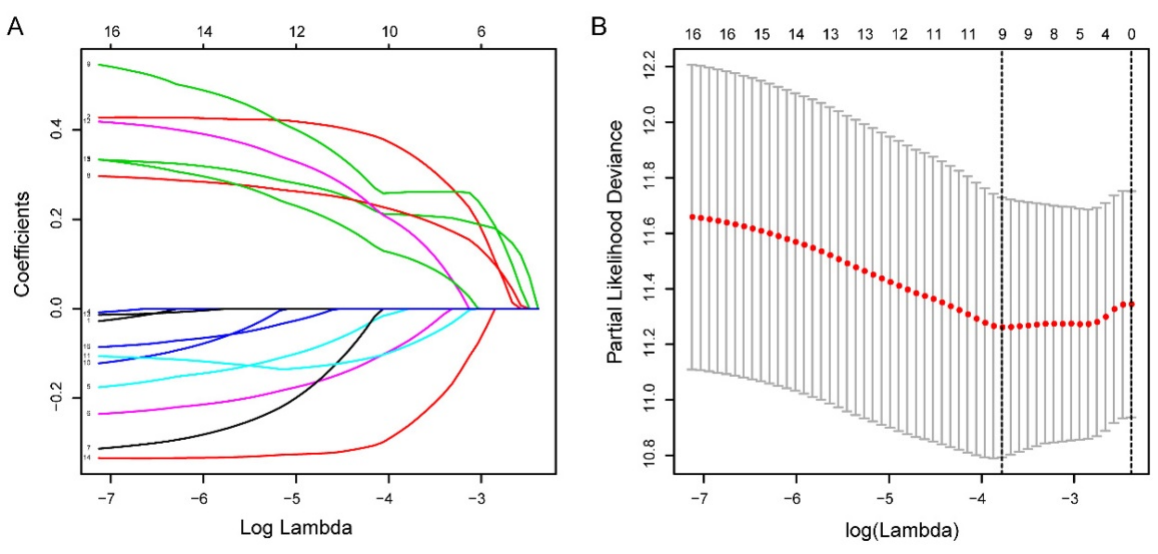
**Identification of prognostic glycolysis-related lncRNAs using LASSO and cox regression analysis. (A)** Plots of the cross-validation error rates. Each dot represents a lambda value along with error bars to give a confidence interval for the cross-validated error rate. **(B)** LASSO coefficient profiles of the lncRNAs associated with the overall survival of endometrial cancer.

**Figure 4:**
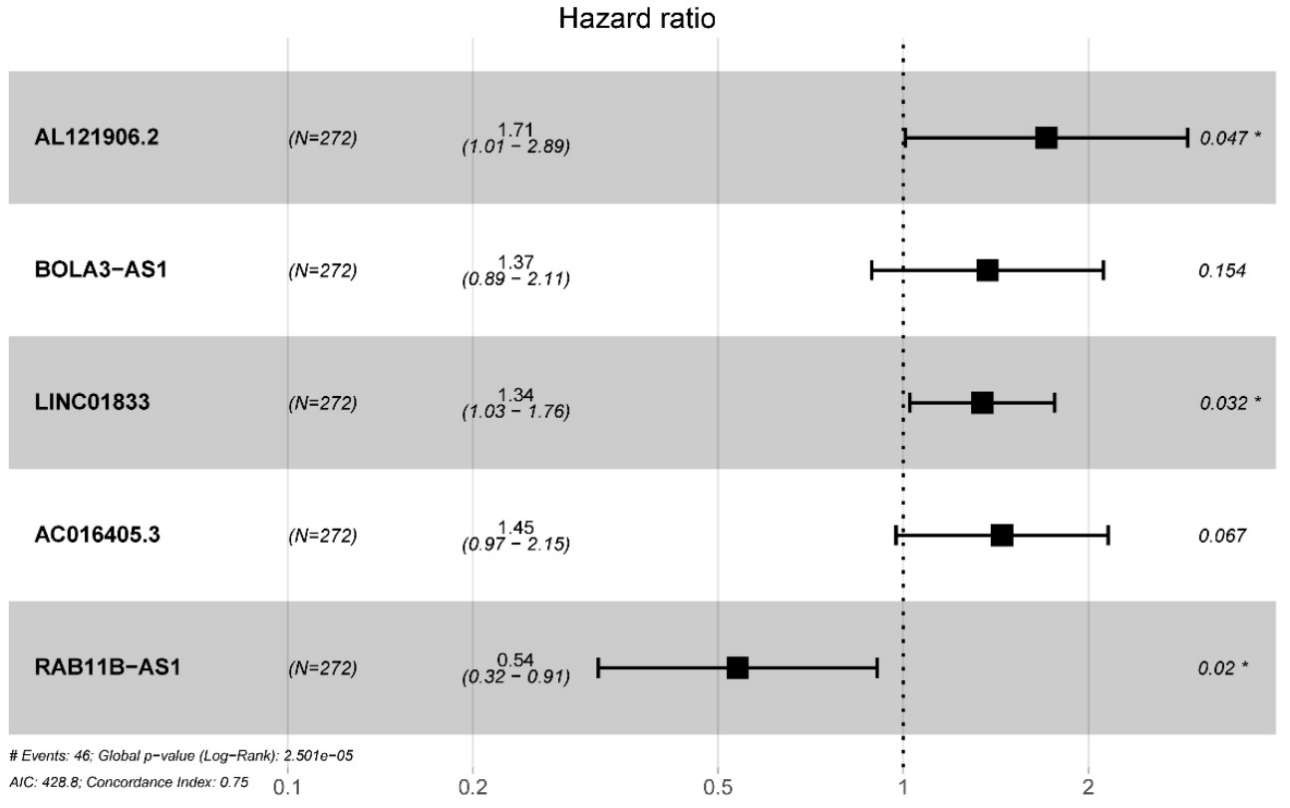
Multivariate cox regression identified 5 prognostic lncRNAs in the training cohort.

Totally 541 TCGA EC patients were divided into the training cohort (n = 272) and the testing cohort (n = 269). Lasso regression analysis identified 9 lncRNA (Figure 3A-B) and multi-variate cox analysis narrowed into 5 lncRNA significantly correlated with prognosis, which are AL121906.2, BOLA3-AS1, LINC01833, AC016405.3, and RAB11B-AS1 (Figure 4 and Table 2). According to multivariate Cox regression analysis results, we constructed a prognostic risk signature as follows: risk score = (0.535559 × expression value of AL121906.2) + (0.315295 × expression value of BOLA3-AS1) + (0.295732× expression value of LINC01833) + (0.369982 × expression value of AC016405.3) + (-0.61956 × expression value of RAB11B-AS1).

**Figure 5:**
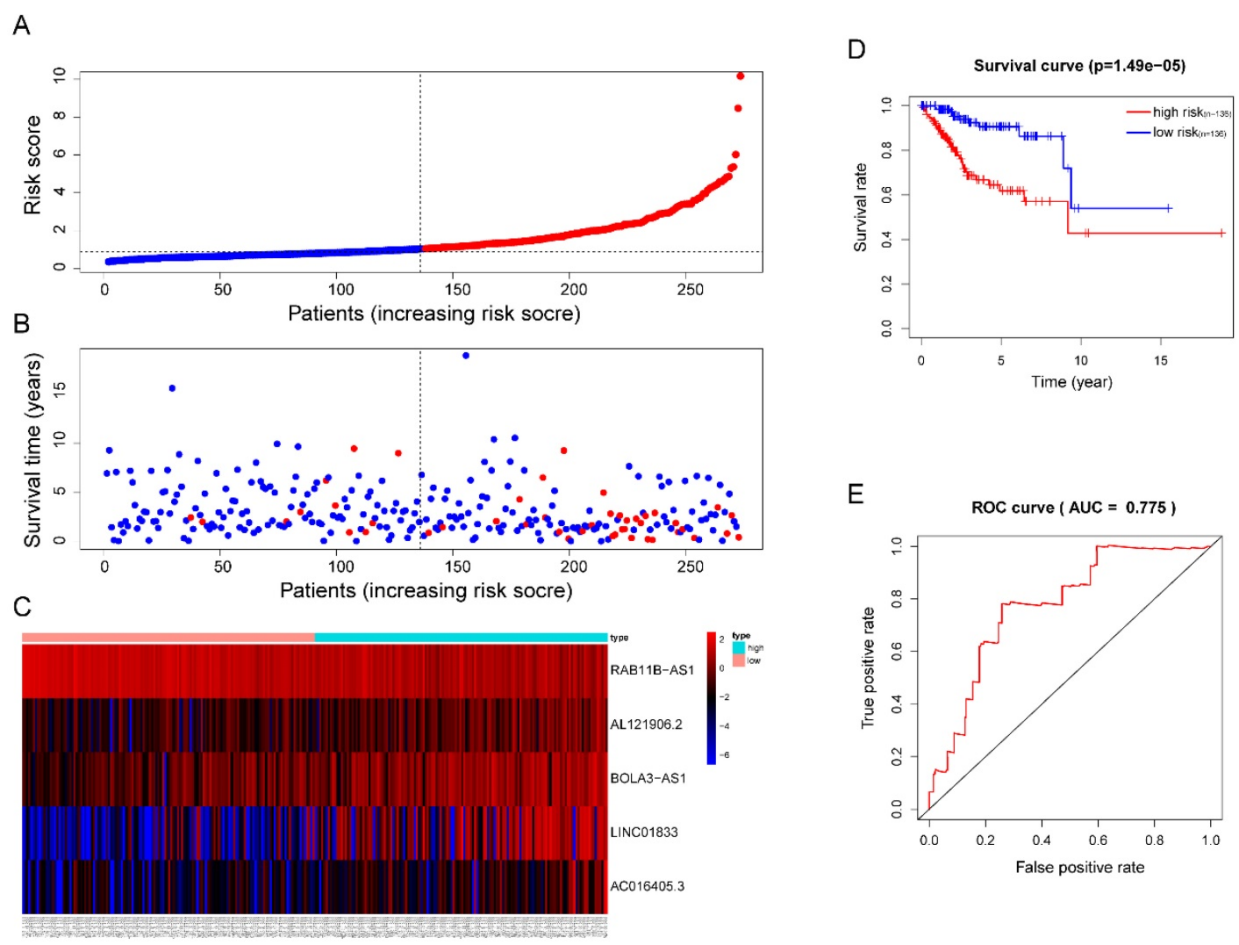
**Prognostic analysis of the lncRNA signature in the TCGA training cohort. (A)**The risk score, **(B)** survival status, **(C)** expression heatmap, **(D)** Kaplan-Meier survival, and **(E)** time-dependent ROC curves of the prognostic model for the TCGA EC training cohort. In part **(A)** and **(B)**, red and blue represent dead and alive, respectively; In part **(C)**, red and blue indicate higher expression and lower expression, respectively.

Then, each patients' risk score was calculated and ranked. Patients were then divided into high-risk (n=136) and low-risk (n=136) subgroups based on the mean risk score. In addition, each individual's risk score distribution and survival status were also ranked (Figure 5A-B). Clearly, patients in the high-risk subgroup were accompanied by more death events and Kaplan-Meier (KM) curve analysis confirmed this result since a significant discrepant OS between both subgroups was observed. The high-risk group showed worse outcomes in comparison with the low-risk subgroup (P =1.49e-05) (Figure 5D). The area under the ROC curve (AUC) of the risk signature in the training cohort was also calculated and the result was 0.775 (Figure 5E). Interestingly, we also observed significant overexpression of AL121906.2, BOLA3-AS1, LINC01833, AC016405.3, and downregulation of RAB11B-AS1 in the high-risk subgroup (Figure 5C).

### Validation of the efficacy of the 5 lncRNA prognostic model

**Figure 6:**
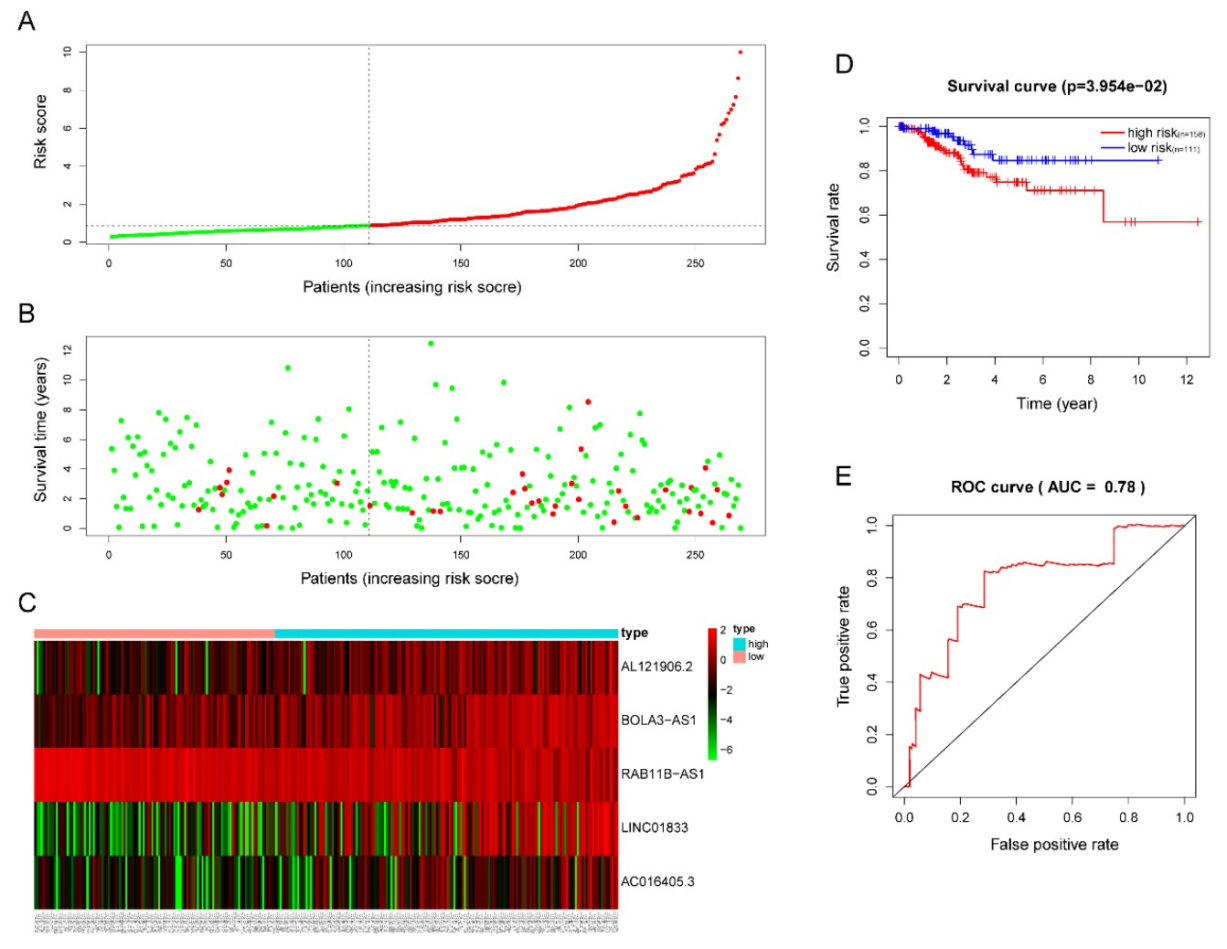
**Validation of the efficacy of the risk signature in the TCGA testing cohort. (A)** The risk score, **(B)** survival status, **(C)** expression heatmap, **(D)** Kaplan-Meier survival, and **(E)** time-dependent ROC curves of the prognostic model for the TCGA EC testing cohort. In part **(A)** and **(B)**, red and green represent dead and alive, respectively; In part **(C)**, red and green indicate higher expression and lower expression, respectively.

**Figure 7:**
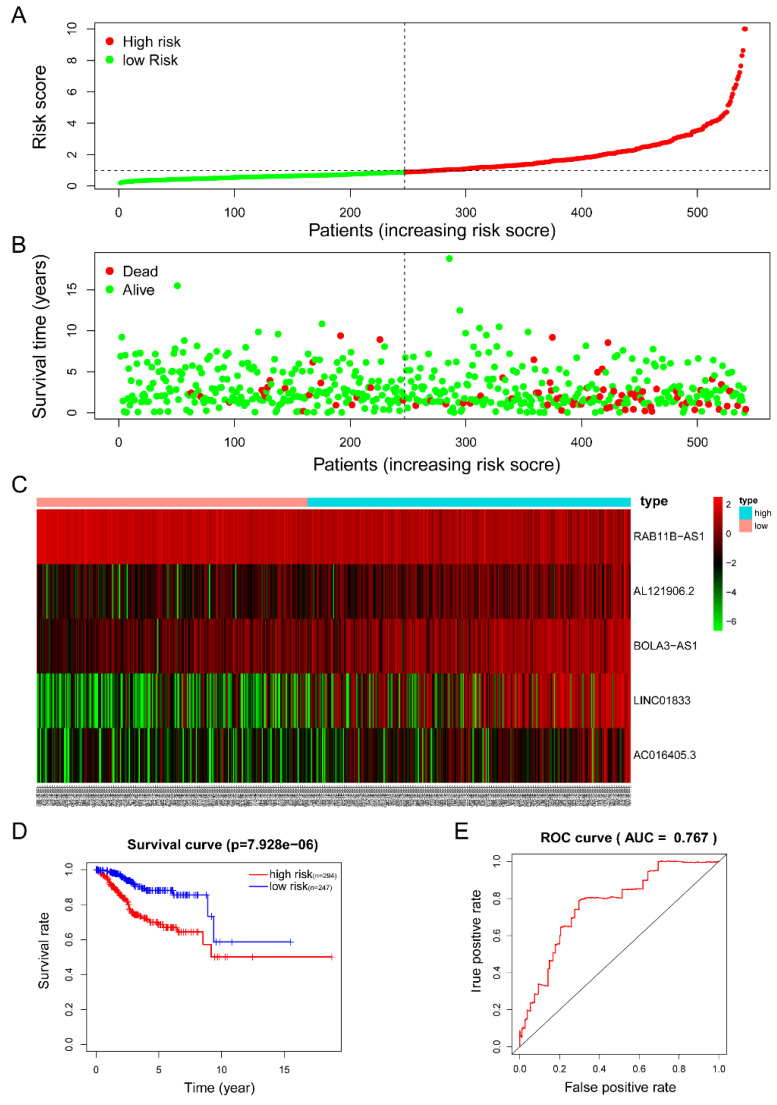
** Estimation of the efficacy of the risk signature in TCGA entire EC cohort. (A)** The risk score, **(B)** survival status, **(C)** expression heatmap, **(D)** Kaplan-Meier survival, and **(E)** time-dependent ROC curves of the prognostic model for the TCGA EC entire cohort. In part **(A)** and **(B)**, red and green represent dead and alive, respectively; In part **(C)**, red and green indicate higher expression and lower expression, respectively.

**Figure 8:**
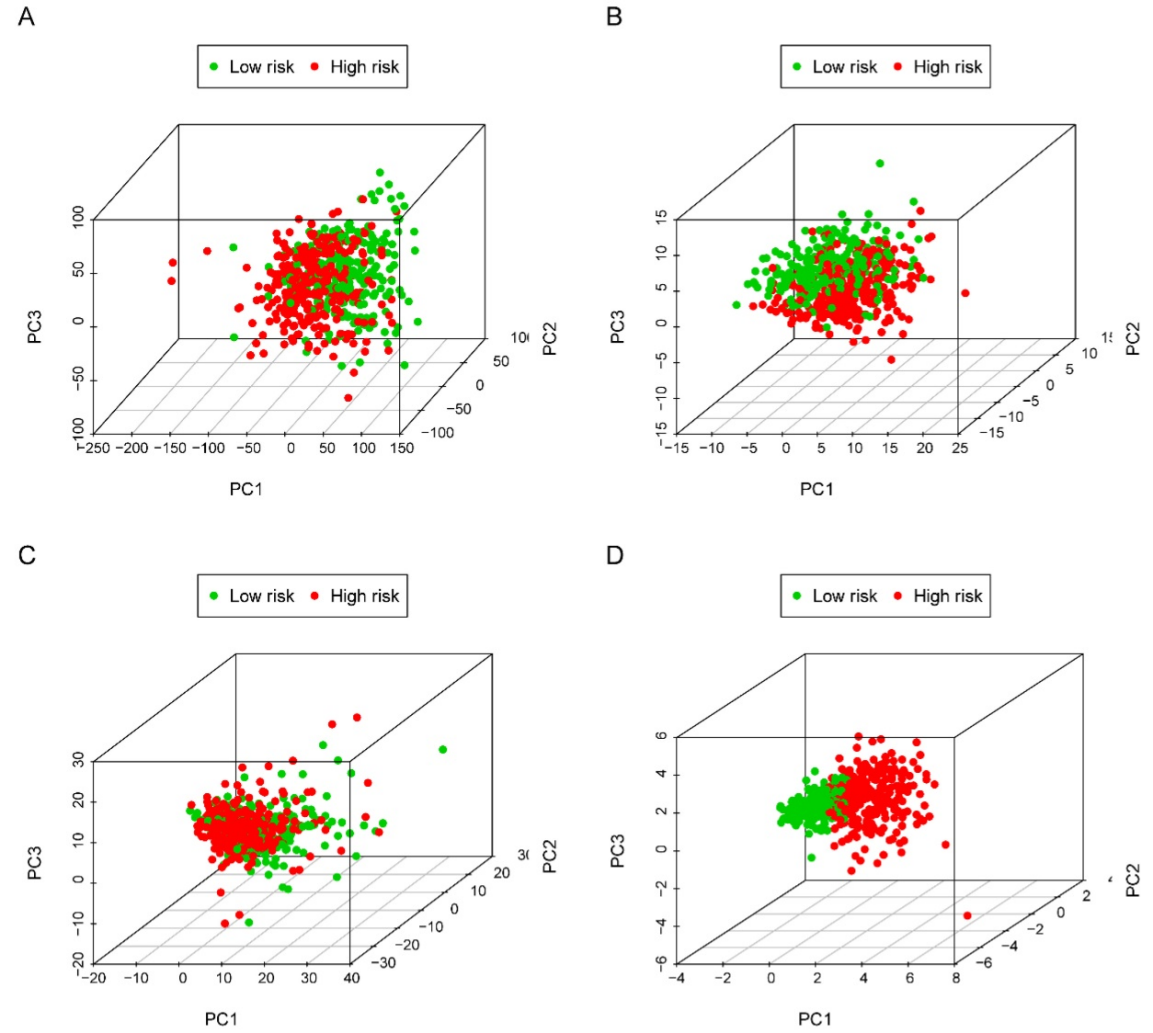
** Principal component analysis (PCA)** of all genes, glycolysis-related genes, glycolysis-related lncRNAs and the risk signature in identifying high and low-risk subgroups. **(A)** all genes; (B) glycolysis-related genes; **(C)** glycolysis-related gene associated lncRNAs and **(D)** risk signature.

The five-lncRNA prognostic model was then brought into the testing and the entire cohort and patients' risk score was calculated based on the formula. Similarly, according to the cut-off value (training cohort's median risk score), each patient was then ranked and categorized into high-risk (n=158, n=294) and low-risk (n=111, n=247) subgroups in the testing cohort and entire cohort, respectively. The efficacy of the model was perfectly validated in the testing and entire cohort since the survival condition and hub lncRNAs expression level in both subgroups showed significant divergence (Figure 6A-C, Figure 7A-C). Survival status distribution and KM analysis showed higher 5-year survival rates in low-risk group patients compared with the high-risk group (P =3.954e-02 and 7.928e-06, respectively) (Figure 6D and 7D). ROC curve analysis showed the AUC of the risk signature was 0.78 and 0.767, respectively (Figure 6E and 7E). PCA analysis displayed a much better-classifying capability of risk signature (Figure 8D) in comparison with all genes (Figure 8A), glycolysis-related genes (Figure 8B), and glycolysis-relate-gene associated lncRNA (Figure 8C).

### Validation of the expression levels of the 5 lncRNA in clinical samples

**Figure 9:**
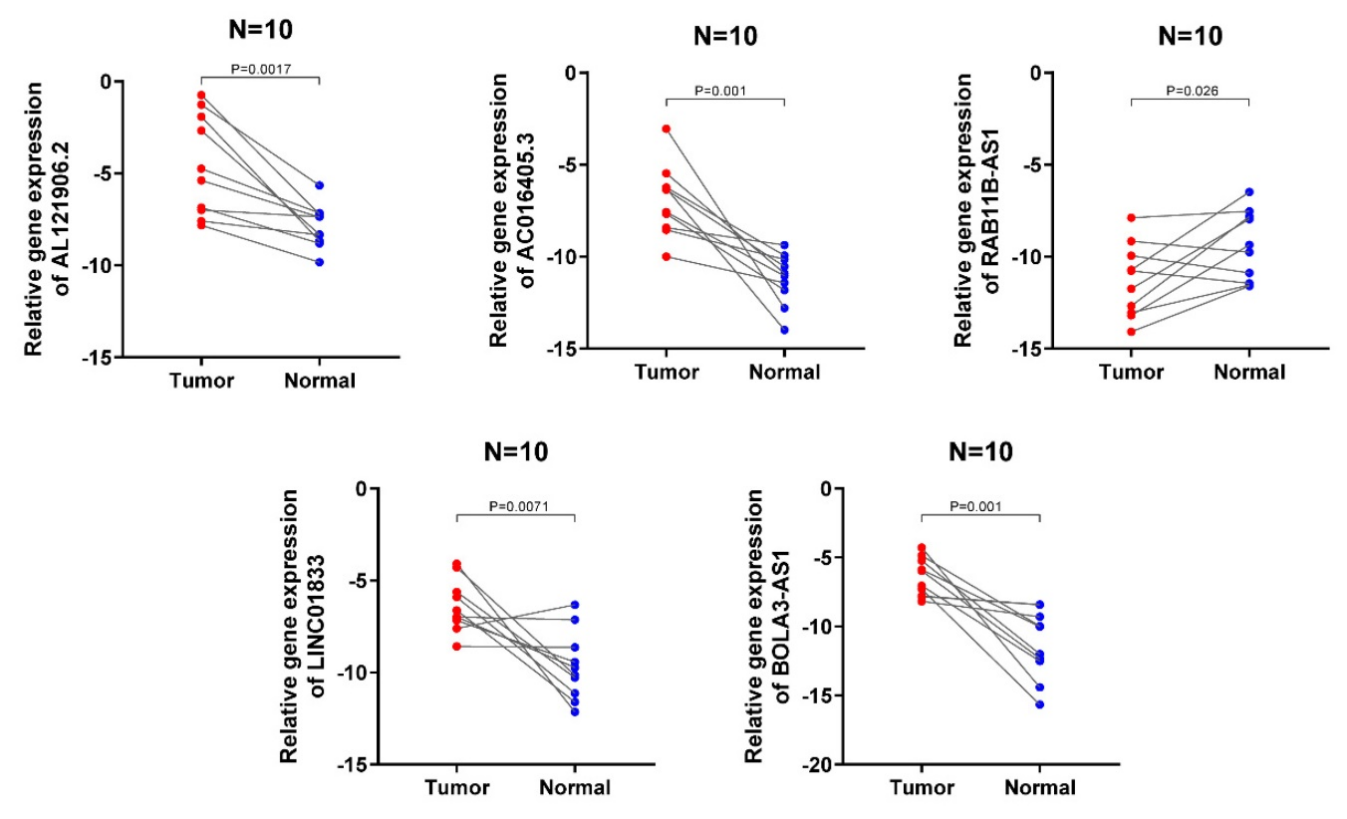
Validation of the expression signature of 5 lncRNA in tissues by qRT-PCR. The student's t-test (two-tailed) was used for the comparative analyses and the significance threshold was set at 0.05 for each test.

The expression signatures of the 5 lncRNA were then investigated in 10 cancerous and normal endometrial clinical specimens. The results showed that AL121906.2, BOLA3-AS1, LINC01833, and AC016405.3 gene levels were upregulated in cancerous tissues, while RAB11B-AS1 was downregulated, which was consistent with the above findings (Figure 9).

### The clinical independence and correlation estimation of the risk signature

**Figure 10:**
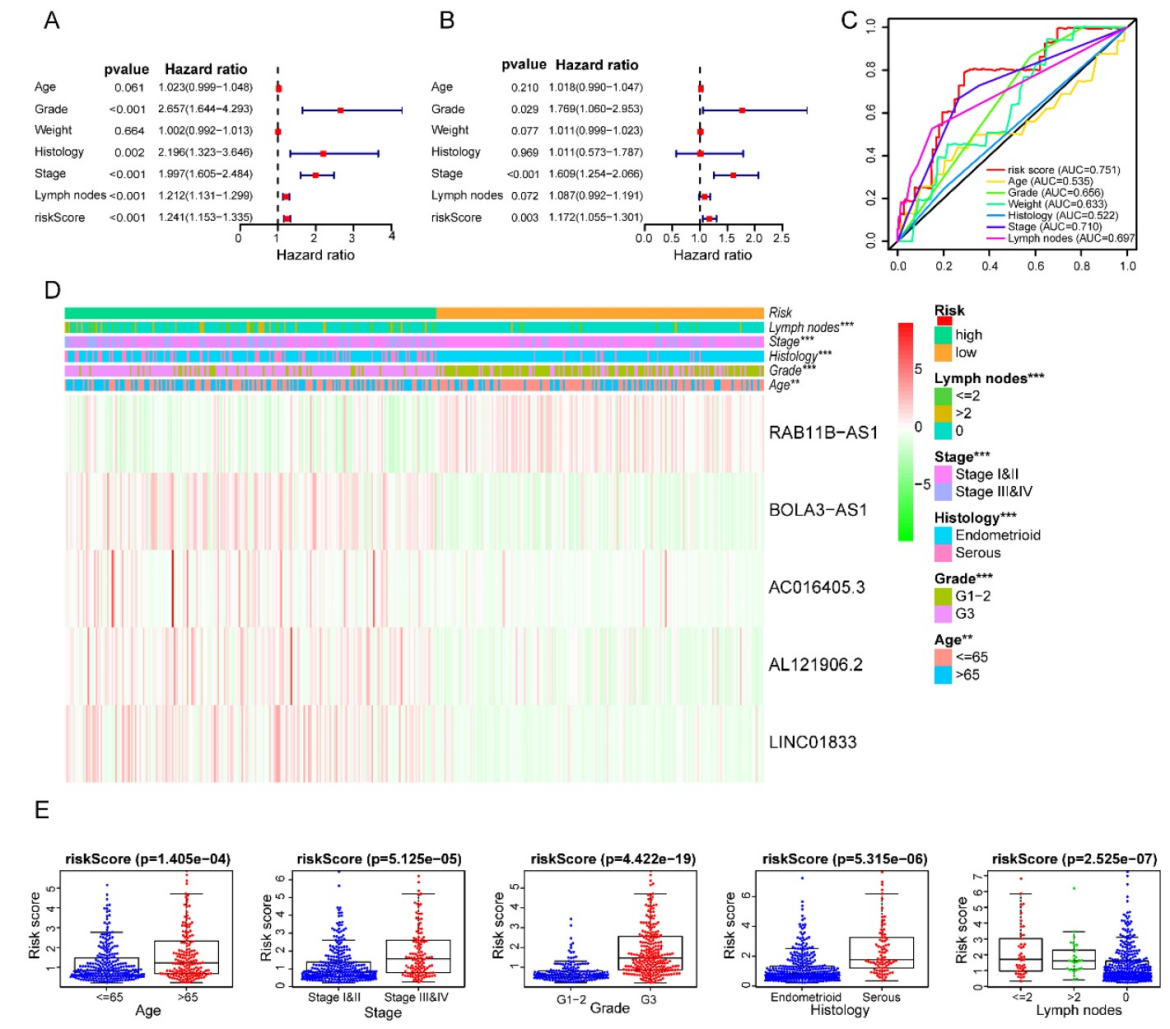
** Clinical characteristics of the prognostic lncRNA signature.** Univariate **(A)** and multivariate **(B)** regression analysis, as well as time-dependent ROC curve analysis **(C)** of the prognostic value between the risk model and EC patients' OS status when compared to or combined with clinical factors; **(D)** Heat map showing the expression of 5 lncRNAs in the risk model and the clinicopathological features of patients with EC; **(E)** Clinicopathological significance of the prognostic signature of endometrial cancer. Red and green indicate higher expression and lower expression, respectively.

**Figure 11:**
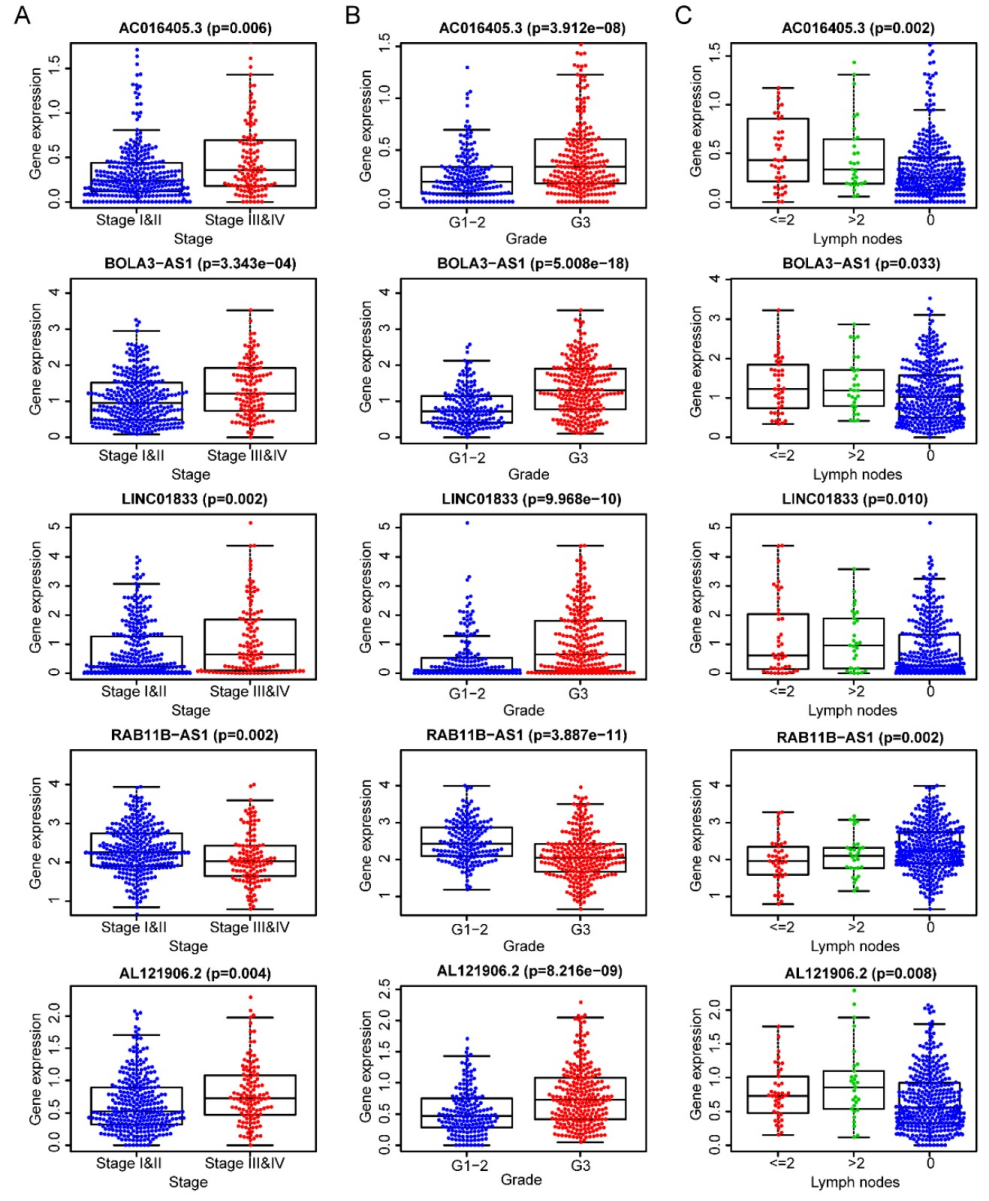
The five lncRNAs are significantly correlated to patients' clinical-stage **(A)**, tumor grade **(B)**, and lymph node numbers **(C)**.

**Figure 12:**
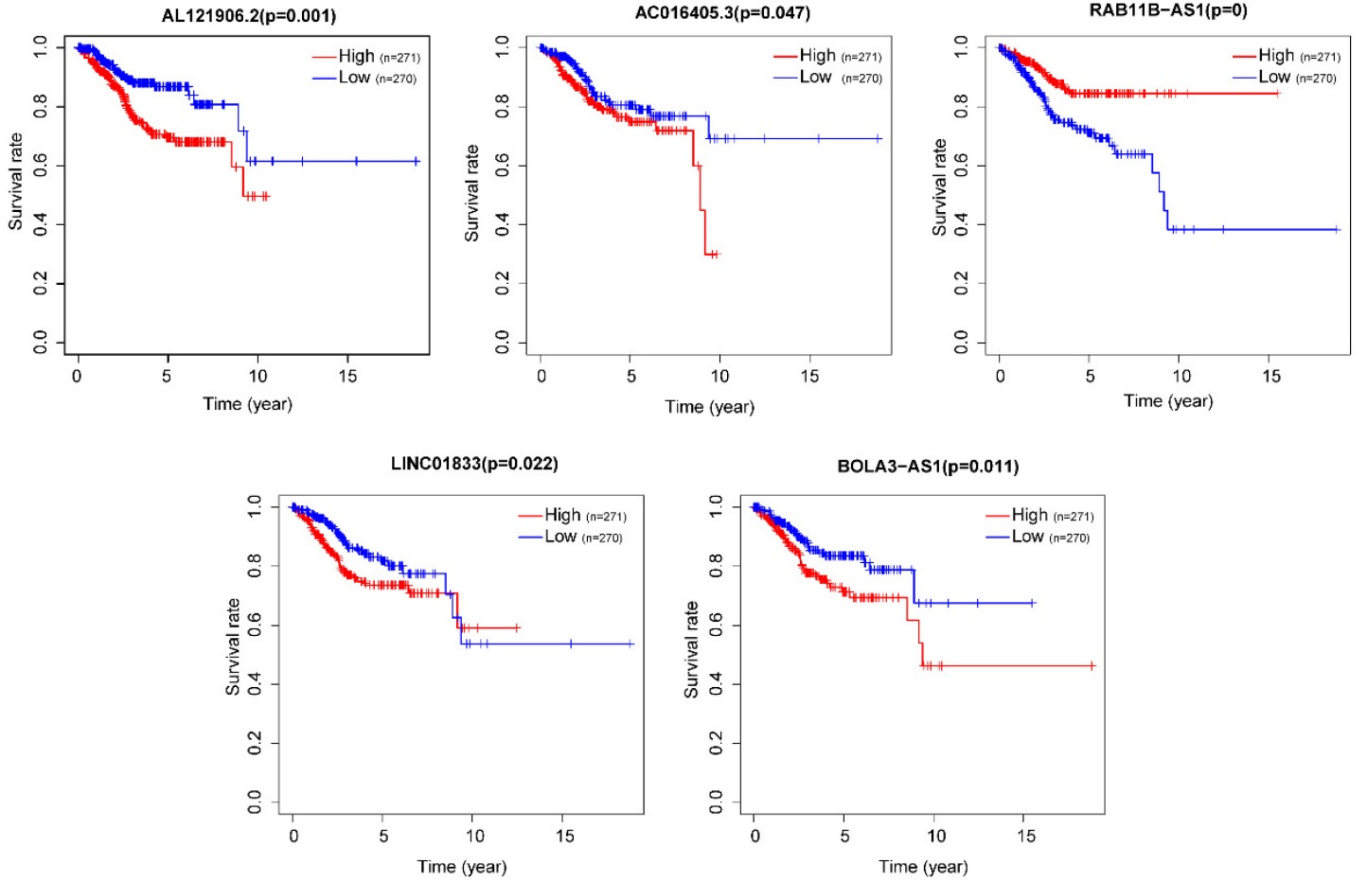
The survival curve of each lncRNA in the risk signature.

After removing 110 TCGA-UCEC patients who lacked a detailed clinical index, we retained 431 patients' gene expression signature and clinical information (Table S2). Then, we integrated the risk model with several clinical factors including weight, stage, histology, and lymph node status, subsequently performing uni- and multivariate analysis to assess the independence of the risk model. Both the uni- and multivariate analysis results presented the model serves as an independent prognostic indicator (P<0.001 and =0.003, respectively) (Figure 10A-B). The AUC value of the prognostic model was 0.751, significantly more precise than clinical index including age (0.535) and weight (0.633), stage (0.710), grade (0.656) and histology (0.522), as well as lymph node status (0.697) (Figure 10C). The clinical features and five lncRNA expression profiles of both risk subgroups' EC patients were combined and displayed in the heatmap (Figure 10D). The distribution of the clinical features was in high concordance with the risk signature. High-risk subgroup patients were more prone to live with older age, advanced stage, poor differentiation, serous tumor, and a larger amount of metastatic lymph nodes (Figure 10D-E). The correlation between each lncRNA from the prognostic model and the patients' clinical features were also measured. All five lncRNAs were shown to be significantly associated with patients' clinical stage, tumor grade, and lymph node numbers (Figure 11A-C). Each lncRNA's survival curve was also drawn and presented in Figure 12.

### Comprehensive nomogram building and evaluation

**Figure 13:**
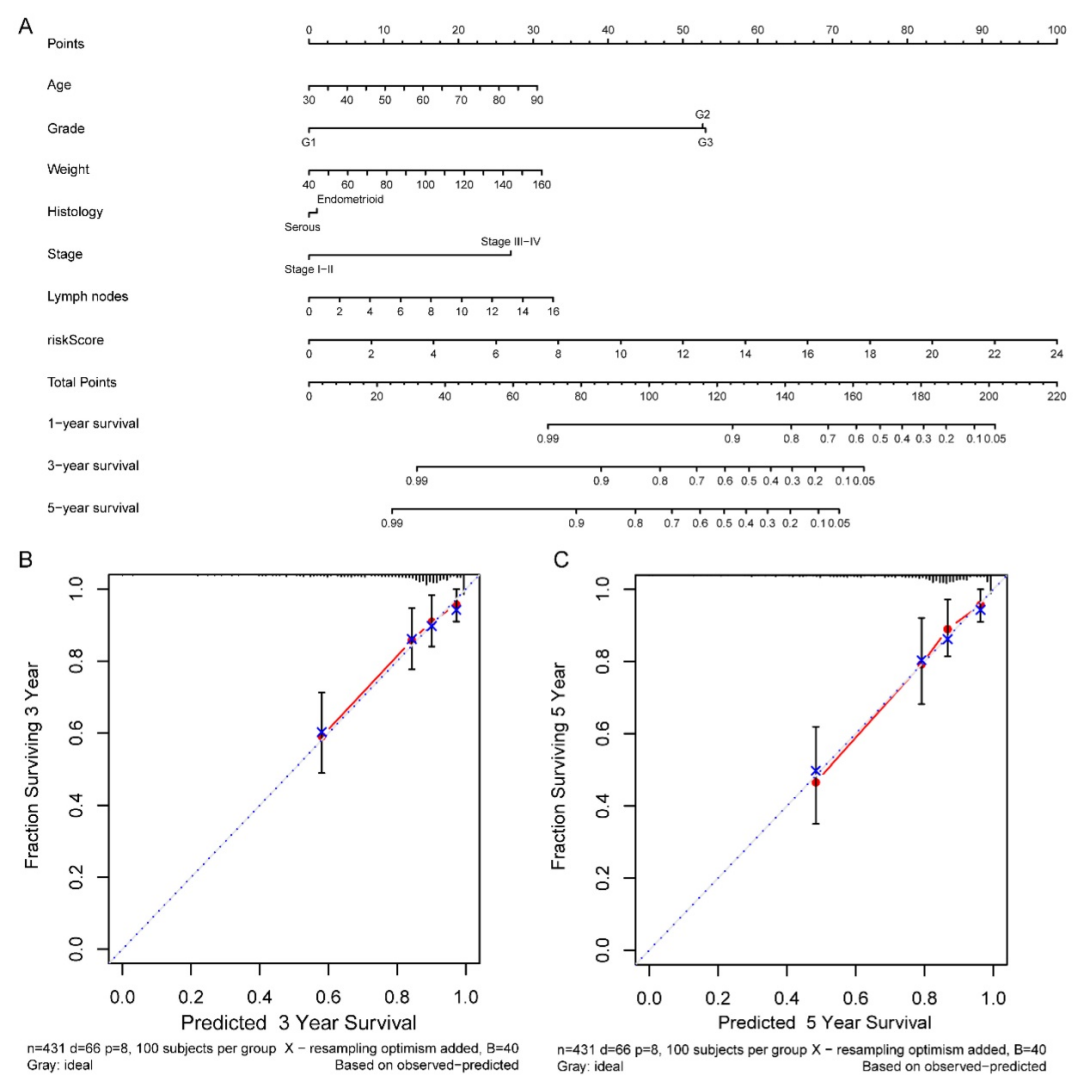
Nomogram for predicting the 5-year survival probability of patients with EC. (A) Prognostic nomogram for EC patients; (B) Calibration curves for the nomogram at 3-, and 5-year.

According to the comprehensive landscape of the integrated patients' risk scores and clinical factors, we built a nomogram predicting EC patients' 5-year survival probability. Seven prognostic parameters, including the lncRNA risk signature and age, grade, weight, histology, stage as well as positive lymph node numbers, were fitted into the nomogram (Figure 13A). Calibration plots demonstrated a high degree of consistency between the actual observation and nomogram forecast in terms of the 3- and 5-year survival rates (Figure 13B-C).

## Discussion

lncRNA has been reported to play a crucial role in various cancer development and progression^12^. Recently, many studies have focused on the value of lncRNA as minimally invasive biomarkers for diagnosis, prognosis, or monitoring curative effects^13^. In addition, the lncRNA-based prognostic model in predicting cancer patients' outcomes has also been widely performed, including hepatocellular carcinoma(HCC), neuroblastoma, and clear cell renal cell carcinoma(ccRCC)^14^-^16^. Liu et al. identified the four-lncRNA risk model as a reliable prognostic and predictive tool for survival prediction in ccRCC. Meng et al. analyzed two GEO neuroblastoma datasets and constructed a four prognostic-related lncRNA signature that accurately predicted the spontaneous regression of neuroblastoma. Similarly, Zhang et al. constructed an immune‐related lncRNA model of TCGA HCC patients for predicting survival and immune checkpoint.

In this study, we analyzed the gene profile of TCGA UCEC patients and identified 36 glycolysis-related lncRNA associated with EC patients' prognosis. Through LASSO and multivariate cox regression analysis, we further screened out 4 onco-lncRNA and 1 antionco-lncRNA that significantly associated with the survival and other clinical characteristics of EC patients. Among the 5 metabolic lncRNA, RAB11B-AS1 and AC016405.3 have already been reported participating in tumor development and progression^17^-^20^. Following our findings that lncRNA RAB11B-AS1 was negatively correlated to the malignant phenotypes in EC, Chen et al. reported the expression of lncRNA RAB11B-AS1 was significantly down-regulated in osteosarcoma. RAB11B-AS1 suppressed the expression of RAB11B, induced the inhibiting effect in cancer cell proliferation^17^. However, Liu et al. found that overexpression of RAB11B-AS1 was significantly associated with a poorer overall survival rate in lung cancer^19^. Likewise, Niu et al. found that hypoxia-induced lncRNA RAB11B-AS1 overexpression could significantly promote the angiogenesis and distant metastasis of breast cancer^18^. As for AC016405.3. Ren et al reported that it was downregulated in glioblastoma tissue. AC016405.3 modulated TET2 expression by sponging of miR-19a-5p, suppressing cell proliferation, and metastasis in glioblastoma^20^. Speak of the heterogeneity of malignancies, more researches are needed to explore the definite mechanisms of these lncRNA in endometrial cancer despite the discrepant results in other cancer types. Note that there has been a rare report on BOLA3-AS1, LINC01833, and AL121906.2 in EC to date. BOLA3-AS1 is the divergent transcript of BOLA3, firstly identified by Fagerberg and his colleagues^21^. It was located in the chr2:74147981-74152389, with 4409 bp total size. LINC01833, also names RP11-89, was identified aberrantly expressed in lung adenocarcinoma and closely related to the Wnt pathway^22^. Therefore, exploring their roles in tumorigenesis may contribute to demonstrating their oncogenic or suppressor function in EC patients.

Note that, the identified 5 glycolysis-related lncRNA successfully divided each of the three independent cohorts into the high-risk and low-risk subgroups with significantly different survival outcomes. Besides, the prognostic role of the five-lncRNA signature is also independent with other well-established clinical risk factors. Last but not least, the comprehensive nomogram that combined the clinical features with the lncRNA risk signature exhibited a precise prognosis calculation model and robust predictive efficacy. As no comprehensive analysis to explore lncRNA profiling has been performed in EC so far, our results strongly highlight the use of this five-lncRNA model as a clinical biomarker for risk stratification and guidance for therapy.

Our research also has certain insufficiencies. First of all, the 5 glycolysis-related lncRNA signature was only constructed and validated in the TCGA UCEC dataset, the robustness of this risk signature and the nomogram upon prognostic prediction need to be further verified in large prospective clinical trials. Secondly, more evidence is needed for demonstrating the deep relationship between EC prognosis and the five lncRNA signatures since rare experimental data are available on these lncRNA. More basic researches should be performed to investigate the potential biological mechanism in EC. Despite the above limitations, our findings still presented a consistent and significant correlation between the risk signature and TCGA UCEC prognosis in both datasets, providing a high level of confidence regarding this signature and nomogram.

## Conclusion

In conclusion, we identified 36 prognostic glycolysis-related lncRNA and constructed a 5-lncRNA risk signature in the TCGA EC cohort. The signature was validated to predict the outcome of TCGA EC patients. Combined with the risk model with other clinical features, the comprehensive nomogram effectively predicted the 5-year survival status of EC patients. These results might offer a new perspective for EC research and individual treatment in clinical practice.

## Supplementary Material

Supplementary figure S1.

Supplementary tables.

## Author Contributions

Yuan Jiang, Rong Li and Jie Chen designed the project. Xianghong Zhu and Jingxian Ling contributed to data analysis and prepared the main manuscript. Pinping Jiang, Huaijun Zhou and Xiaoqiu Tang revised and submitted the manuscript. All authors reviewed the manuscript.

## Data availability statement

The expression data were retrieved from the TCGA database and the clinical information was downloaded from the cBioPortal website. Please contact the author for data and materials requests.

## Figures and Tables

**Table 1:** Univariate Cox regression identified 36 glycolysis-related lncRNAs correlated to endometrial cancer patients' OS

Gene ID	HR	HR.95L	HR.95H	P-value
LINC01852	0.488690394	0.178375738	1.338849689	0.1637793
AC009237.14	1.48581723	1.118644428	1.973507206	0.006254431
AL121906.2	2.095111272	1.31992591	3.32555881	0.001704205
AC137630.1	0.470801715	0.208857058	1.06127251	0.069284431
AL391244.3	1.428564194	0.83448683	2.445569641	0.193492956
BOLA3-AS1	2.037341702	1.395449508	2.974497598	0.000228019
LINC02321	1.672816412	0.977459144	2.862845743	0.060545553
LNCTAM34A	0.582077699	0.373908319	0.906143112	0.016556345
AL390195.1	0.365138911	0.119892458	1.11205014	0.076219776
AC005256.1	0.703284433	0.463265941	1.067656718	0.098411961
KRT7-AS	1.242167381	0.882702258	1.748018414	0.213443788
AC074117.1	1.763036878	0.827640976	3.755612787	0.141655021
AC046143.1	1.889683098	0.706785866	5.05231129	0.204674046
AC027319.1	0.326628514	0.143390022	0.744027966	0.007724145
AC137630.3	0.647699414	0.427088828	0.982265289	0.040935569
AC009005.1	0.873874393	0.683692362	1.116959174	0.281638559
AC107057.1	1.341348491	1.010954462	1.779719899	0.041802903
AC013724.1	0.819595126	0.633263084	1.060753718	0.130590302
AC104825.1	0.735741866	0.497646308	1.087752656	0.123971342
AC092171.2	1.367524038	0.899092072	2.080011662	0.143512032
LINC01833	1.568669071	1.225766494	2.007497078	0.000346899
BX322234.1	2.521058226	1.472964444	4.31492736	0.000745144
AC068987.4	1.494046027	1.050977114	2.12390308	0.02528676
AL390195.2	0.555143274	0.244704194	1.259414682	0.159096459
HNF1A-AS1	0.575483591	0.36875966	0.898095424	0.014962893
AC083799.1	0.801226216	0.526846148	1.218502691	0.300175168
AC016405.3	1.671240165	1.179277899	2.368435543	0.003890094
AL353622.1	0.691810656	0.48171986	0.993527615	0.04602914
AC068134.2	0.712169609	0.459159461	1.104595668	0.12958385
AL513318.2	1.18639764	0.791319263	1.778725005	0.408117466
RAB11B-AS1	0.50027757	0.305254977	0.819897023	0.005999678
AC026202.2	0.436092823	0.175859113	1.08141652	0.073286494
LINC00324	0.558919428	0.274310372	1.138822876	0.109157698
AC010980.2	2.095253825	1.27285558	3.449007616	0.003629227
SRP14-AS1	0.568934195	0.283122605	1.143271901	0.113209489
AL353747.2	0.668291486	0.44848395	0.995829415	0.047647242

Abbreviation: OS: overall survival; HR: Hazard ratio.

**Table 2:** Multivariate Cox regression selected 5 glycolysis-related lncRNAs correlated to endometrial cancer patients' OS

Gene ID	HR	HR.95L	HR.95H	P-value
AL121906.2	1.708402794	1.008273081	2.894692083	0.046526287
BOLA3-AS1	1.370663326	0.888333176	2.114879871	0.154197608
LINC01833	1.344109231	1.025345947	1.761970806	0.032258754
AC016405.3	1.447708934	0.973886125	2.152059779	0.067377932
RAB11B-AS1	0.538180705	0.319521992	0.90647429	0.019853954

Abbreviation: OS: overall survival; HR: Hazard ratio.

## Abbreviation

EC: endometrial cancer
UCEC: Uterine Corpus Endometrial Carcinoma
TCGA: The Cancer Genome Atlas
ROC: Receiver operating characteristic
AUC: area under the curve
LASSO: Least Absolute Shrinkage and Selection Operator
OS: overall survival
FC: fold change
MSigDB: Molecular Signatures Database
PCA: principal component analysis
