# Supplementary figures and images for: Construction of a Glycolysis-related long noncoding RNA signature for predicting survival in endometrial cancer
Source: J Cancer. 2021 Jan 1;12(5):1431–44. doi: 10.7150/jca.50413 (PMC7847640; doi:10.7150/jca.50413)

**Figure S1.** Clinical characteristics of each prognostic lncRNAs from the signature.

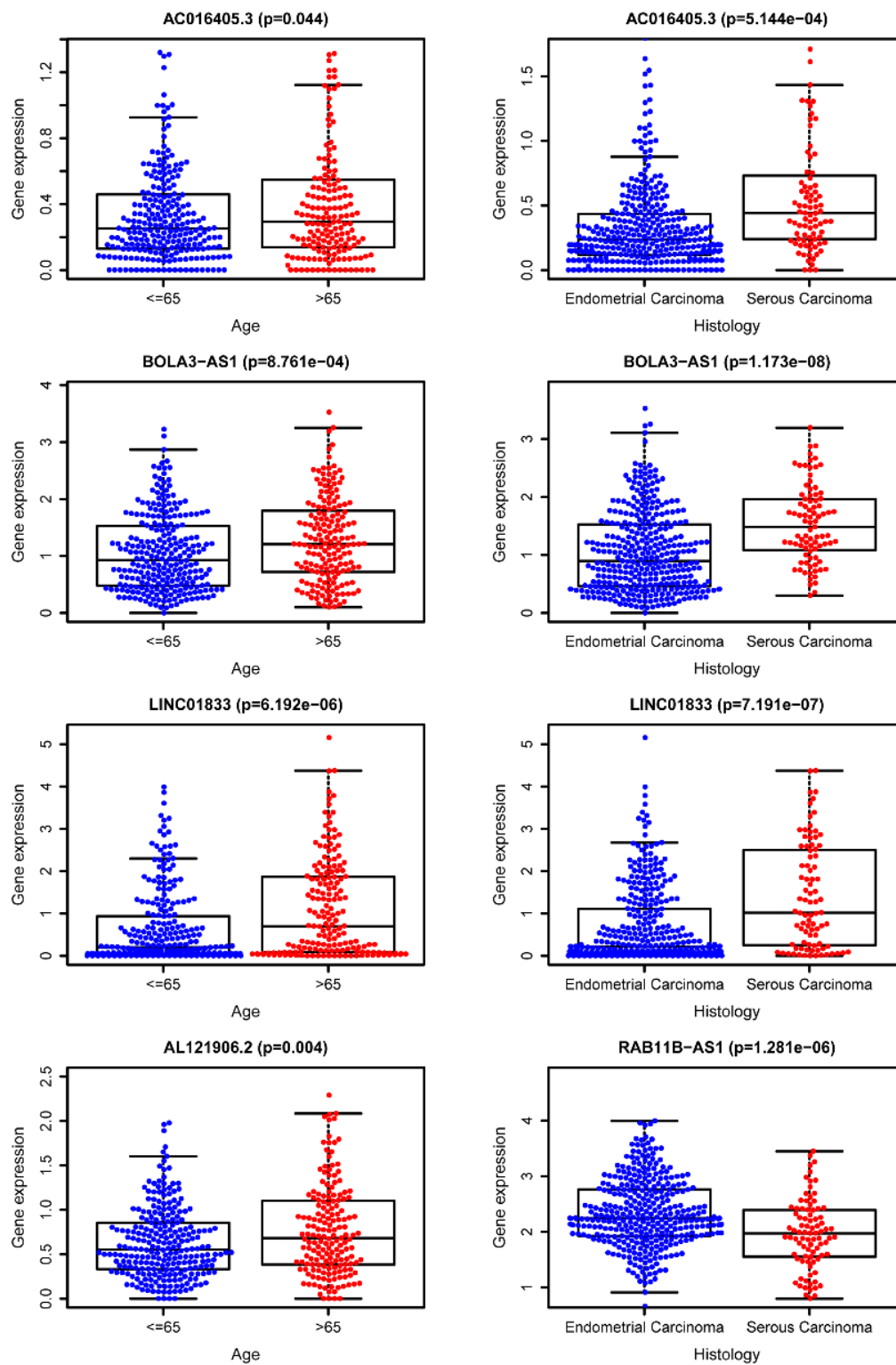

Supplement: Supplementary file 1 — Supplementary figure S1. [file jcav12p1431s1.pdf]
